# Effects of dietary *Lactobacillus plantarum* and AHL lactonase on the control of *Aeromonas hydrophila* infection in tilapia

**DOI:** 10.1002/mbo3.362

**Published:** 2016-04-20

**Authors:** Wenshu Liu, Chao Ran, Zhi Liu, Qian Gao, Shude Xu, Einar Ringø, Reidar Myklebust, Zemao Gu, Zhigang Zhou

**Affiliations:** ^1^ Key Laboratory of Freshwater Biodiversity Conservation and UtilizationMinistry of AgricultureCollege of FisheriesHuazhong Agricultural UniversityWuhanHubei430070China; ^2^ Key Laboratory for Feed Biotechnology of the Ministry of AgricultureFeed Research InstituteChinese Academy of Agricultural SciencesBeijing100081China; ^3^ Institute of Animal Husbandry and VeterinaryJiangxi Academy of Agricultural ScienceNanchangJiangxi330200China; ^4^ College of Fisheries and Life ScienceShanghai Ocean UniversityShanghaiChina; ^5^ Faculty of Biosciences, Fisheries and EconomicsNorwegian College of Fishery ScienceUiT The Arctic University of NorwayTromsøNorway; ^6^ Institute of Anatomy and Cell BiologyUniversity of BergenBergenNorway

**Keywords:** AHL lactonase, disease, immunity, *lactobacillus*, tilapia

## Abstract

This study addressed the effects of dietary *Lactobacillus plantarum* or/and *N*‐acylated homoserine lactonase (AHL lactonase) on controlling *Aeromonas  hydrophila* infection in juvenile hybrid tilapia (*Oreochromis niloticus*♀ × *O. aureus* ♂). Fish were fed *Lb. plantarum* subsp*. plantarum* strain JCM1149 (10^8^ CFU/g feed) or/and AHL lactonase AIO6 (4 U/g) and were exposed to a chronic challenge of *A. hydrophila* NJ‐1 (10^5^ cells/mL) for 14 days. Intestinal (foregut) alkaline phosphatase (IAP) activities were evaluated 1 day post challenge to reflect the resistance of fish against *A. hydrophila* infection. Parallel groups of fish with the same dietary assignments while unchallenged were also included to investigate the effect of dietary *Lb. plantarum* or/and AIO6 supplementation on gut health of tilapia. The results showed that IAP activity was significantly lower in fish fed with diets supplemented with *Lb. plantarum* JCM1149 or the combination of *Lb. plantarum* JCM1149 and AIO6, indicating enhanced resistance against *A. hydrophila*. Light microscopy and transmission electron microscopy images of foregut revealed damage caused by *A. hydrophila* NJ‐1, but dietary *Lb. plantarum* JCM1149 or/and AIO6 significantly alleviated the damages. Compared to the fish immersed in *A. hydrophila* NJ‐1, dietary *Lb. plantarum* JCM1149 or AIO6 could maintain the microvilli length in the foregut of tilapia. However, among the unchallenged groups of fish, the microvilli length in the foregut of tilapia fed AIO6 (singly or combination) and the microvilli density of tilapia fed AIO6 (singly) were significantly lower than those of the control, though the microvilli density in the combination treatment was significantly improved. Additionally, the dietary *Lb. plantarum* JCM1149 could down‐regulate the expression of stress‐related gene in the gut after the acute phase. In conclusion, the dietary *Lb. plantarum* JCM1149 is recommended to control the *A. hydrophila* infection in tilapia.

## Introduction

China has the largest aquaculture production in the world; approximately 55 million tons (m.t.) of aquatic products were harvested in 2012, and this included 41 m.t. of aquaculture products (FAO, 2014). However, the rapid increase in intensive cultivation and the use of nonstandard techniques has lead to dramatic increase in disease outbreaks in the commercial aquaculture industry. In recent years, nearly 20% of the production has been lost through diseases, resulting in economic losses of more than US$ 10 billion (Liu et al. 2014). In particular, infectious diseases caused by *Aeromonas hydrophila* is the major problem (Nielsen et al. 2001; Xiao et al. 2011).

It was common practice for several decades to use chemotherapeutic/antimicrobial agents to prevent and control infectious diseases. However, routine use of these agents leads to adverse consequences, such as development of antibiotic resistance (Hektoen et al. 1995; Aarestrup 2005; Cabello 2006; Rice 2009). Therefore, the scientific community has evaluated alternative strategies to control pathogens of aquatic animals. Two promising groups are probiotics and quenching enzymes, which are gradually to be used in aquaculture.

Probiotics are defined as live microbial supplements that benefit the host (Fuller 1987). In the animal feed industry including aquaculture, probiotics were extensively applied and its beneficial effects were well recognized (Vanbelle et al. 1990). Probiotic interferes with the action of potential pathogens by the production of inhibitory molecules (Fuller 1987) or/and the competition of adhesion sites on the gastrointestinal mucosa (Merrifield et al. 2010; Lazado et al. 2011; Korkea‐Aho et al. 2012; Sharifuzzaman et al. 2014). As an important group of probiotics, LAB strains can locally produce organic acids that lower the pH, excrete natural antimicrobial compounds, or compete with pathogens for nutrients, and adhesion sites (Ringø et al. 2010, a review). In particular, *Lactobacillus* sp. is widely used in aquaculture for disease prevention (Verschuere et al. 2000; Ringø et al. 2010, a review). A previous investigation by Zhou et al. (2012) revealed that *Lactobacillus plantarum* subsp. *plantarum* JCM1149 adhered to intestinal mucosa and decreased mortality of zebrafish (*Danio rerio*) challenged with *A. hydrophila* as the prevention effect. In addition, this strain competed for intestinal adhesion sites with *A. hydrophil*a and alleviated gut mucosa damage caused by *A. hydrophila* in ex vivo tilapia as the control effects (*Oreochromis niloticus*♀ × *O. aureus* ♂) (Ren et al. 2013). Most importantly, pre‐experiment validated the control effects of JCM1149 on tilapia in vivo at 10^8^ cell/g as the optimal dose (detailed data not shown here).

Many bacterial pathogens regulate the expression of virulence factors via quorum‐sensing networks. Therefore, quenching these systems may contribute to the prevention of related diseases. A possible way to interfere with quorum sensing is signal inactivation by enzymatic degradation or modification. For this purpose, AHL lactonases and acylases that hydrolyze *N*‐acylhomoserine lactone (AHL) signaling molecules have been investigated (Fetzner 2015). *Aeromonas hydrophila* possesses an AHL‐dependent quorum‐sensing system (Lynch et al. 2002), and AHL lactonase could hydrolyze quorum‐sensing signal molecules (butaryl‐homoserine lactone and hexanoylhomoserine lactone) of *A. hydrophila* and reduce its virulence (Zhang et al. 2011; Cao et al. 2012; He et al. 2013). The protection effects of quenching enzymes of AHL lactones (e.g., AHL lactonases B565, AIO6, AI‐96, and QsdA) against *A. hydrophila* were verified by Cao et al. (2012) in zebrafish in vivo (AI‐96) and Zhang et al. (2011) in vitro. In a tilapia in vivo trial, fish dietary AIO6 (3.7 U/g diet) showed resistance to *A. hydrophila* (unpublished data).

The detailed study of the diseases and the organisms that provoke them allow for the design of diverse prevention (before infection) and control (during infection) strategies (Singh 2013). However, most studies focused on investigating the prevention effects after treated with probiotics or other supplementation (Newaj‐Fyzul et al. 2014; Van Hai 2015). In this study, we addressed the effects of *Lb. plantarum* JCM1149 in combination with AHL lactonase as a control strategy against *A. hydrophila* infection in tilapia. Their combined effect was also tested considering their different mechanisms of action by evaluating intestinal alkaline phosphatase (IAP) activity, gut morphology, and intestinal immune response of tilapia fed four diets and subjected to chronic challenge of *A. hydrophila*.

## Materials and Methods

### Fish

Juvenile hybrid tilapia (*O. niloticus*♀ × *O. aureus* ♂), with mean body weight ~3.0 g, were obtained from an aquaculture farm in Haikou, Hainan, China. Fish were acclimatized in a recirculating aquarium system for 2 weeks before the feeding trial.

### Bacteria and quenching enzyme

*Lactobacillus plantarum* JCM 1149 was purchased from the Japanese Collection of Microorganisms (Tsukuba, Japan). The bacterium was inoculated on MRS agar plates and incubated at 30°C for 48 h under aerobic conditions; then, one clone was inoculated to MRS broth and incubated at 30°C for 48 h under aerobic condition. The pathogenic strain *A. hydrophila* NJ‐1 was a gift from Dr. Yongjie Liu from Nanjing Agricultural University (Nanjing, China) and it was grown in LB broth. The virulence‐related genes are regulated via quorum‐sensing system in *A. hydrophila* NJ‐1 (Cao et al. 2014). The linear relationship between plate CFU count and optical density was used to estimate the count of JCM1149 and NJ‐1 strain. After growing in medium for 2 days, cells were collected by centrifugation (10 min, 2300*g*, 4°C). The pellet was washed in PBS buffer (pH 7.2) twice, and resuspended in 5 mL of PBS buffer at 10‐fold (10^−1^–10^−6^) dilutions. The optical density at 600 nm (OD_600_) was measured at each dilution, and then, the linear relationship between optical density and bacterial cell concentration was calculated. Commercial AHL lactonase AIO6, with good storage stability (Zhang et al. 2011), was obtained from Cuimiesu (Challenge Group, Beijing, China).

The ingredients for basal diet were supplied by Tangshan Jiayuan Feed Co., Ltd (Tangshan, China). All ingredients that were ground into fine powder through a 200‐mm mesh were thoroughly mixed with JCM 1149 cells and/or AHL lactonase AIO6. Then, an appropriate volume of water was added to produce a stiff dough. The dough was then pelleted with a handle noodle machine and air‐dried for 24 h at room temperature. The JCM1149 cells were added into the water together with skimmed milk (as the protective agent), and similar volumes of skimmed milk were added to all diets. Thereafter, the pellets were smashed into pieces, and then proper particles were collected by sieving. The basal diet was comprised of 47% fish meal, 24% soybean meal, 24% wheat flour, 2% soybean oil, and 3% premix (Cao et al. 2014). The basal diet was used as control (CK). Three supplemental diets were prepared, that is, the control diet, the enzyme diet supplemented with 4.0 U/g of AIO6 (A) (Cao et al. 2014), the probiotics diet supplemented with 10^8^ CFU/g of *Lb. plantarum* (L) (Zhou et al. 2012), and the combined diet supplemented with 4.0 U/g of AIO6 and 10^8 ^CFU/g of *Lb. plantarum* (C). The viable cells in each diet were counted at the first, third, and seventh day. The number of *Lactobacillus* decreased to some degree at the seventh day. Therefore, the diets were prepared once a week and stored at 4°C. The stability of AIO6 in the experimental diets was assessed as previously described (Cao et al. 2014).

### In vivo exposure to bacterial pathogen

The experimental treatments are shown in Table 1. One hundred and twenty fish were randomly distributed into eight tanks (15 fish per tank). Each diet was randomly assigned to two tanks. When feeding was started, *A. hydrophila* NJ‐1 was added to the rearing water at concentration of 10^5^ cfu/mL in treatments CK+, L+, A+, and C+ (Table 1) every second day. Fish were hand fed to apparent satiation twice daily (09:00 and 15:00) for 2 weeks. The fish readily ate the experimental diet with no difference from the control. The experimental protocol was performed in accordance to the guidelines approved by the Animal Ethics Committee of the Feed Research Institute, Chinese Academy of Agricultural Sciences (2012‐ZZG‐ZF‐001).

**Table 1:** Experimental treatments fed with different diets either subjected to *Aeromonas hydrophila* challenge or not

Treatments	Diets	Immersed in
CK	Basal feed	
CK+	Basal feed	*A. hydrophila* NJ‐110^5^ cells/mL
L	Basal feed containing *Lactobacillus plantarum* at 10^8^ cfu/g feed	
L+	Basal feed containing *Lb. plantarum* at 10^8^ cfu/g feed	*A. hydrophila* NJ‐110^5^ cells/mL
A	Basal feed containing AHL lactonase at 4.0 U/g feed	
A+	Basal feed containing AHL lactonase at 4.0 U/g feed	*A. hydrophila* NJ‐110^5^ cells/mL
C	Basal feed containing *Lb. plantarum* at 10^8^ cfu + AHL lactonase at 4.0 U/g feed	
C+	Basal feed containing *Lb. plantarum* at 10^8^ cfu + AHL lactonase at 4.0 U/g feed	*A. hydrophila* NJ‐110^5^ cells/mL

### Sampling

After feeding for 1, 3, 5, and 7 days, three fish were randomly sampled from each treatment and dissected under MS222 (25 mg/L) anesthesia. Based on the results of Ren et al. (2013), the foregut (proximal part of the intestine) was sampled. Immediately after sampling, the gut segments were rinsed two times with PBS buffer, and stored at −80°C until further use. After feeding for 2 weeks, three fish were sampled and the foregut was rinsed with PBS buffer carefully and fixed in 2.5% glutaraldehyde for morphology evaluation.

### IAP activity analysis

IAP activity of foregut samples after 1 day of feeding was quantified as reported (Bates et al. 2007), which was used to assess the number of *A. hydrophila* adhered to the gut. Briefly, intestinal samples of three fish from two tanks, six fish per treatment were pooled, weighed, homogenized, and incubated in *p*‐nitrophenyl phosphate liquid substrate system (Sigma‐Aldrich Trading Co. Ltd, Shanghai, China) for 30 min. Afterwards, the absorbance was measured at 405 nm using a microtiter plate reader (Multiskan MK3; Thermo, Marietta, OH, USA). *p*‐nitrophenol (PNP) was used as standard. The linear relation between alkaline phosphatase and OD_405_ was calculated. The standard curve equation was *y* = 2.883*x* − 0.123 (*R* ^2^ = 0.994) and the examination range was between 0 and 2.70.

### Gut morphology evaluation

Foregut segments for light microscopy (LM), scanning electron microscopy (SEM), and transmission electron microscopy (TEM) analysis were evaluated as previously described (Ringø et al., 2007; Salma et al. 2011). The samples were randomly numbered, and the images were taken by the operator in molecular imaging center at the University of Bergen. To assess the effect of different treatments on foregut morphology, 10 LM or TEM images were randomly selected from each fish in each treatment. The impacts of treatments were monitored in terms of; abnormal lamina propria, disorganized microvilli, disintegrated tight junctions, loosening of enterocytes from basal membrane, edema, and number of goblet cell. The morphological changes were scored as follows: 0 = not observed, 1 = low (1–3 out of 10 images), 2 = moderate (4–6 out of 10 images), and 3 = high (7 or more out of 10 images) (Ringø et al., 2007; Salma et al. 2011). The selection and evaluation of microscope images was carried out by Dr. Wenshu Liu without bias.

### Intestinal cytokine genes expression

A previous study by Cao et al. (2014) investigated the effect of dietary AHL lactonase on intestinal cytokine genes expression of fish, which recommended that expression of *A. hydrophila* infection, intestinal cytokine genes expression should be evaluated only in the *Lb. plantarum* groups. The expression of pro‐inflammatory *il‐1β*,* tnf‐α*, anti‐inflammatory *tgf‐β*, and stress marker *hsp70* in the foregut of tilapia was assessed after 1, 3, 5, and 7 day of feeding according to Liu et al. (2014). Total RNA was extracted using a TRIzon Reagent RNA kit (Promega, Mannheim, Germany), and RNA quality was analyzed by visualization on a 1.2% agarose gel. RNA was dissolved in 50 *μ*L RNase‐free water and stored at −80°C until use. *c*DNA was synthesized for quantitative reverse‐transcription PCR (RT‐*q*PCR) using the Rever Tra Ace‐*α*‐RT‐PCR kit (TOYOBO, Shanghai, China). The *q*PCR primers were referenced from Liu et al. (2013). Additional dissociation curve analysis was performed and showed a single melting curve in all cases. The *q*PCR was performed with the SYBR Green Premixes Ex Taq TMII (TaKaRa, Beijing, China) in an iQ5 multicolor real‐time PCR Detection system (Bio‐Rad, Beijing, China). The total volume of the PCR reactions was 20 *μ*L and consisted of: 10 *μ*L SYBR Green Premix Ex TaqII (2×), 1 *μ*L primer of each, 2 *μ*L *c*DNA, and 6 *μ*L distilled/deionized H_2_O. The cycling conditions were as follows: 95°C for 3 min and then 40 cycles of 95°C for 20 sec, 58°C for 20 sec, and 72°C for 20 sec. All *q*PCRs were performed at least three times. Data analysis was conducted using the 2^−ΔΔCT^ method (Livak and Schmittgen 2001) and the *β*‐actin gene was chosen as the internal standard.

### Statistical analysis

Results are expressed as the mean ± SD. Differences between treatments were determined using a one‐way analysis of variance with the SPSS Version 17.0 (SPSS Inc., Chicago, USA) statistical software package. Significant differences were accepted at *P *<* *0.05.

## Results

### Intestinal morphology

**Figure 1:**
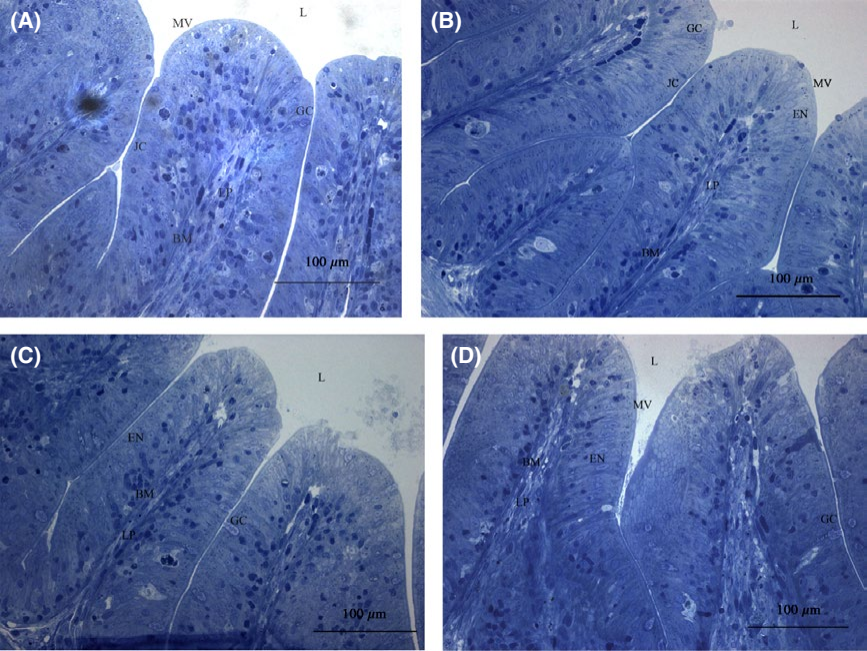
Light microscopy (LM) of the foregut of tilapia from CK (A), L (B), A (C), and C (D) treatment. (*L*, lumen; *MV*, microvilli; *LP*, lamina propria; *JC*, junctional complex; *GC*, goblet cells; *EN*, enterocyte nucleus; BM, basal membrane). CK, fish dietary basal diet; L, fish dietary *Lactobacillus plantarum*; A+, fish dietary AHL lactonase; C+, fish dietary *Lb. plantarum* and AHL lactonase.

**Figure 2:**
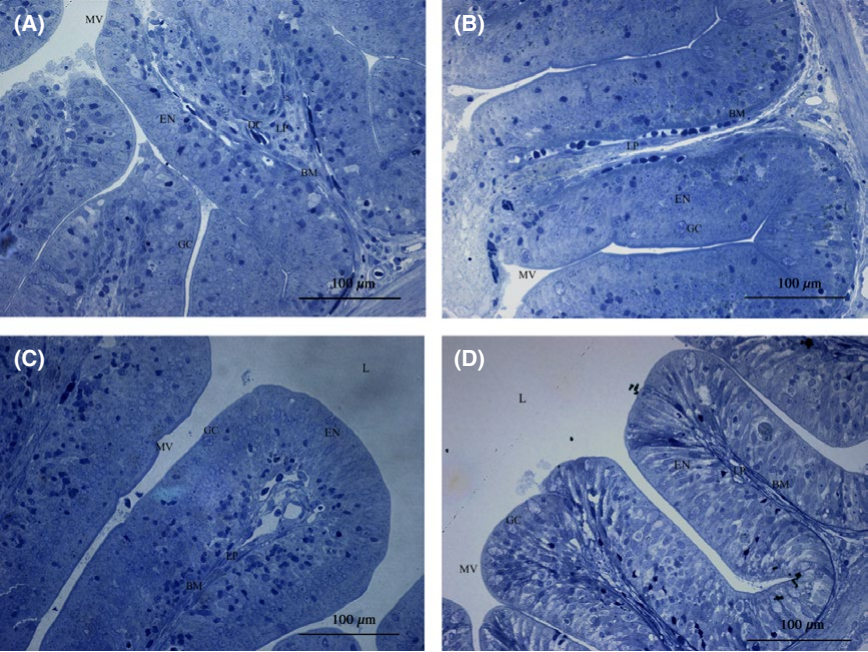
Light microscopy (LM) of the foregut of tilapia from CK+ (A), L+ (B), A+ (C), and C+ (D) treatment. (*L*, lumen; *MV*, microvilli; *LP*, lamina propria; *JC*, junctional complex; *GC*, goblet cells; *EN*, enterocyte nucleus; *BM*, basal membrane; *DC*, Dead cell). CK+, fish dietary basal diet and immersed by *A. hydrophila* NJ‐1; L, fish dietary *Lactobacillus plantarum* and immersed by *A. hydrophila* NJ‐1; A+, fish dietary AHL lactonase and immersed by *A. hydrophila* NJ‐1; C+, fish dietary *Lb. plantarum* and AHL lactonase and immersed by *A. hydrophila* NJ‐1.

**Figure 3:**
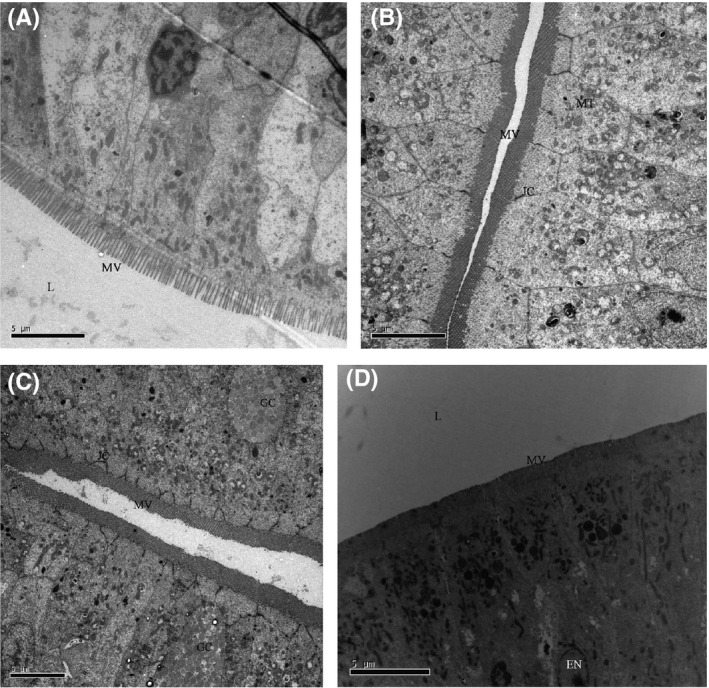
Transmission electron microscopy (TEM) of the foregut of tilapia from CK (A), L (B), A (C), and C (D) treatment.(*L*, lumen; *MV*, microvilli; *JC*, junctional complex; *GC*, goblet cells; *MT*, mitochonadria). CK, fish dietary basal diet; L, fish dietary *Lactobacillus plantarum*; A+, fish dietary AHL lactonase; C+, fish dietary *Lb. plantarum* and AHL lactonase.

**Figure 4:**
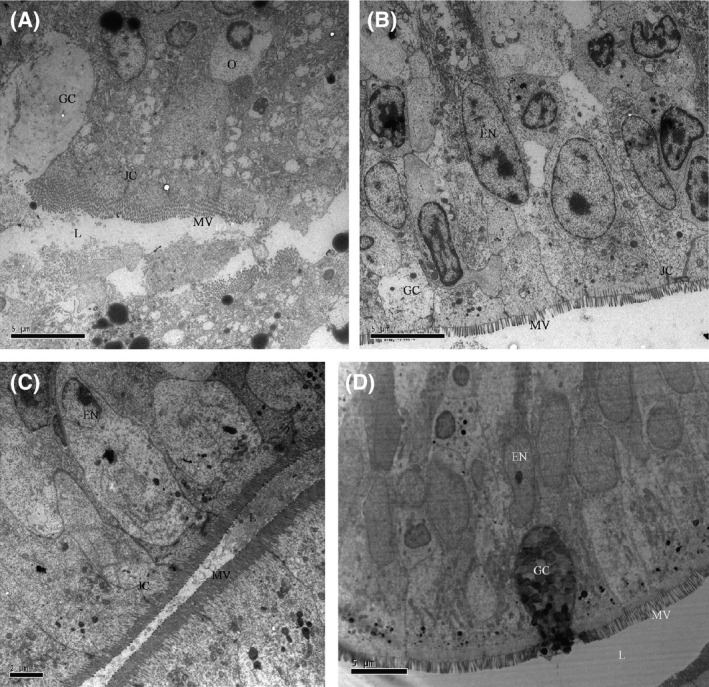
Transmission electron microscopy (TEM) of the foregut of tilapia from CK+ (A), L+ (B), A+ (C), and C+ (D) treatment.(*L*, lumen; *MV*, microvilli; *JC*, junctional complex; *GC*, goblet cells). CK+, fish dietary basal diet and immersed by *A. hydrophila* NJ‐1; L, fish dietary *Lactobacillus plantarum* and immersed by *A. hydrophila* NJ‐1; A+, fish dietary AHL lactonase and immersed by *A. hydrophila* NJ‐1; C+, fish dietary *Lb. plantarum* and AHL lactonase and immersed by *A. hydrophila* NJ‐1.

**Figure 5:**
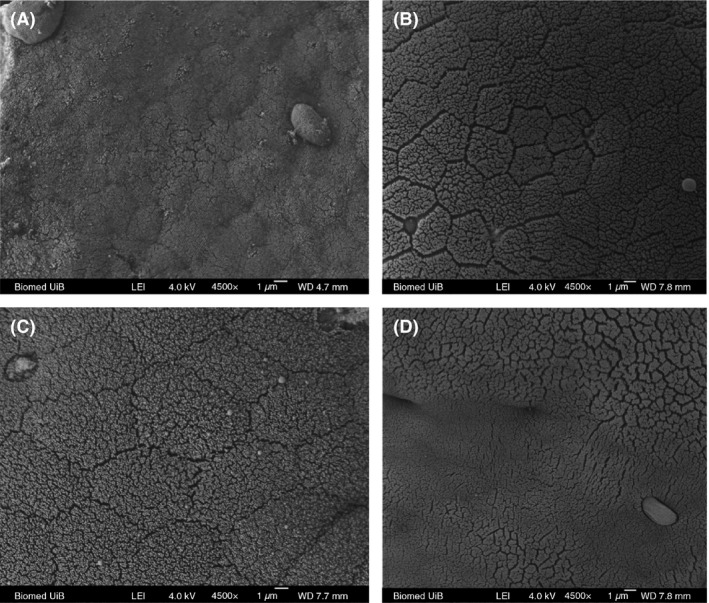
Scanning electron microscopy (TEM) of the foregut of tilapia from CK (A), L (B), A (C), and C (D) treatment.(*L*, lumen; *MV*, microvilli; *JC*, junctional complex; *GC*, goblet cells; *MT*, mitochonadria). CK, fish dietary basal diet; L, fish dietary *Lb. plantarum*; A+, fish dietary AHL lactonase; C+, fish dietary *Lb. plantarum* and AHL lactonase.

**Figure 6:**
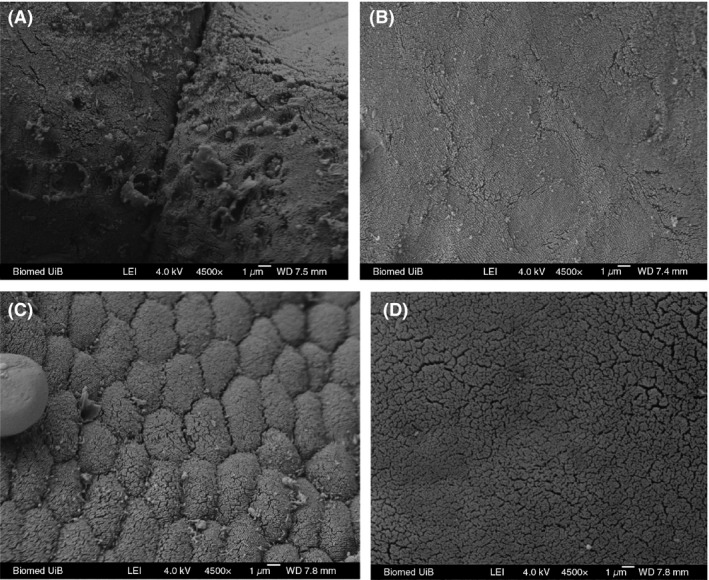
Scanning electron microscopy (SEM) of foregut of tilapia from CK+ (A), L+ (B), A+ (C), and C+ (D) treatment. CK+, fish dietary basal diet and immersed by *Aeromonas hydrophila* NJ‐1; L, fish dietary *Lactobacillus plantarum* and immersed by *A. hydrophila* NJ‐1; A+, fish dietary AHL lactonase and immersed by *A. hydrophila* NJ‐1; C+, fish dietary *Lb. plantarum* and AHL lactonase and immersed by *A. hydrophila* NJ‐1.

LM (Fig. 1) and TEM (Fig. 3) images showed that the appearance of enterocytes in the foregut was normal for group CK, L, A, and C. In addition, the undamaged lamina propria and well‐organized uniform microvilli were observed. However, LM evaluations revealed abnormal lamina propria (increased lamina propria widening and granulocyte number), disintegrated tight junctions, increased number of goblet cell, loosening of enterocytes from basal membrane and cell debris in lumen in the CK+ treatment (Fig. 2). In treatment groups L+, A+, and C+, *Lb. plantarum* or/and AHL lactonase alleviated the histology damages caused by *A. hydrophila* (Fig. 2B, C, and D). TEM images revealed normal intestinal histology of groups CK, L, A, and C (Fig. 3). However, TEM evaluations revealed severe damage was observed in the foregut of CK+, indicated by disorganized microvilli, loss of microvilli, disintegrated tight junctions, increased numbers of goblet cells, and edema (Fig. 4A). In contrast, normal intestinal morphology of groups L+, A+, and C+ was revealed, similar to the CK group (Fig. 4B, C, and D). SEM evaluations revealed normal appearance of intestinal morphology of groups CK, L, A, and C (Fig. 5). Disorganized microvilli was observed in group CK+, but not in groups L+, A+, and C+ (Fig. 6).

A summary of the morphological changes observed in the treatments are presented in Table 2 (LM) and Table 3 (TEM). A general trend was noticed; dietary *Lb. plantarum* or/and AHL lactonase did not significantly affect the intestinal histology. However, exposure of fish to *A. hydrophila* NJ‐1 resulted in cell debris in the lumen, and disorganization of the microvilli, but dietary *Lb. plantarum* or/and AHL lactonase significantly alleviate these damages.

**Table 2:** Intestinal (foregut) morphological changes in different groups of tilapia observed by light microscopy at the end of 14 feeding days

Morp hology	Under normal status				Under infected status			
	CK	L	A	C	CK+	L+	A+	C+
Presence of cell debris in lumen	0	0	0	1	1	0.5	1	0
Disorganized microvilli	0	0	0	0	2	0	0	0
Abnormal lamina propria	0	0	1	0	2.5	1	0	0
Number of goblet cells	0	0	1.5	1	3	1.5	1	1
Disintegrated tight junctions	0	0	0	0	1.5	0	0	0
Loosening of enterocytes from basal membrane	0	0	0	0	1.5	2	0	0
Column totals	0	0	2.5	2	11^x^	5^y^	2^z^	1^z^

CK, fish fed basal diet; L, fish dietary lactobacillus; A, fish dietary AHL; C, fish dietary lactobacillus and AHL; CK+, L+, A+, C+ mean fish dietary different diets under infected status. The morphological changes are based on light microscopy evaluation of 10 micrographs from each treatment group. Tissue changes were assessed as follows: 0 = not observed; 1 = low frequency (1–3 out of 10 images); 2 = moderate frequency (4–6 out of 10 images) and 3 = high frequency (7 or more out of 10 images); Means, *N* = 2 fish. Different superscript letter means significant difference when compared to CK or CK+.

**Table 3:** Intestinal (foregut) morphological changes in different groups of tilapia observed by TEM at the end of 14 feeding days

Morphology	Under normal status				Under infected status			
	CK	L	A	C	CK+	L+	A+	C+
Abnormal lamina propria	0	0	0	0	2	0	0	0
Disorganized microvilli	0	0	0	0	2	0	1	0
Loss of microvillus	0	0	0	0	2	0	1	0
Empty goblet cells	0	0	0	0.5	1.5	0	0	0
Filled goblet cells	1	0.5	2	1	2	1.5	1.5	1.5
Disintegrated tight junctions	0	0	0	0	2	1	0	0
Edema	0	0	0	0	3	0	0	0
Loosening of enterocytes from basal membrane	0	0	0	0	2	0	0	0
Column totals	1^a^	0.5^a^	2^a^	1.5^a^	16.5^x^	2.5^y^	3.5^y^	1.5^y^

CK, fish fed basal diet; L, fish dietary lactobacillus; A, fish dietary AHL; C, fish dietary lactobacillus and AHL; CK+, L+, A+, C+ mean fish dietary different diets under infected status. The morphological changes are based on light microscopy evaluation of 10 micrographs from each treatment group. Tissue changes were assessed as follows: 0 = not observed; 1 = low frequency (1–3 out of 10 images); 2 = moderate frequency (4–6 out of 10 images) and 3 = high frequency (7 or more out of 10 images; Means, *N* = 2 fish. Different superscript letter means significant difference when compared to CK or CK+.

Among the groups challenged with *A. hydrophila*, dietary *Lb. plantarum* or AHL lactonase significantly improved the microvilli length(~1.3 *μ*m) compared with CK+ (~0.8 *μ*m) (Table 4). However, among the unchallenged groups, the microvilli length and density from AHL lactonase group (A) were significantly (*P *<* *0.05) lower than the control group. In the combination group (C), the microvilli length (0.89 *μ*m) was significantly (*P *<* *0.05) lower compared to group CK (1.23 *μ*m), similar to the microvilli length (0.91 *μ*m) in group A. However, the microvilli density significantly (*P *<* *0.05) increased in combination group C (~115/*μ*m^2^) compared to the other treatment groups suggesting potential synergistic effect of *Lb. plantarum* and AHL lactonase on the microvilli density.

**Table 4:** Intestinal (foregut) microvilli length and density in different groups of tilapia by TEM and SEM at the end of 14 feeding days

Morphology	Under normal status				Under infected status			
	CK	L	A	C	CK+	L+	A+	C+
Length (TEM)	1.23 ± 0.05^a^	1.35 ± 0.03^a^	0.91 ± 0.02^b^	0.89 ± 0.03^b^	0.77 ± 0.02^x^	1.32 ± 0.03^y^	1.39 ± 0.11^y^	0.89 ± 0.03^x^
Density (SEM)	71.7 ± 2.50^a^	72.3 ± 4.50^a^	45.3 ± 1.70^b^	114.7 ± 7.04^c^	58.3 ± 1.70^x^	57.7 ± 1.89^x^	61.7 ± 2.87^x^	66.3 ± 3.30^x^

CK, fish fed basal diet; L, fish dietary lactobacillus; A, fish dietary AHL; C, fish dietary lactobacillus and AHL; CK+, L+, A+, C+ mean fish dietary different diets under infected status. Different superscript letter means significant difference when compared to CK or CK+.

### Immune protection estimated by 24 h IAP activity

**Figure 7:**
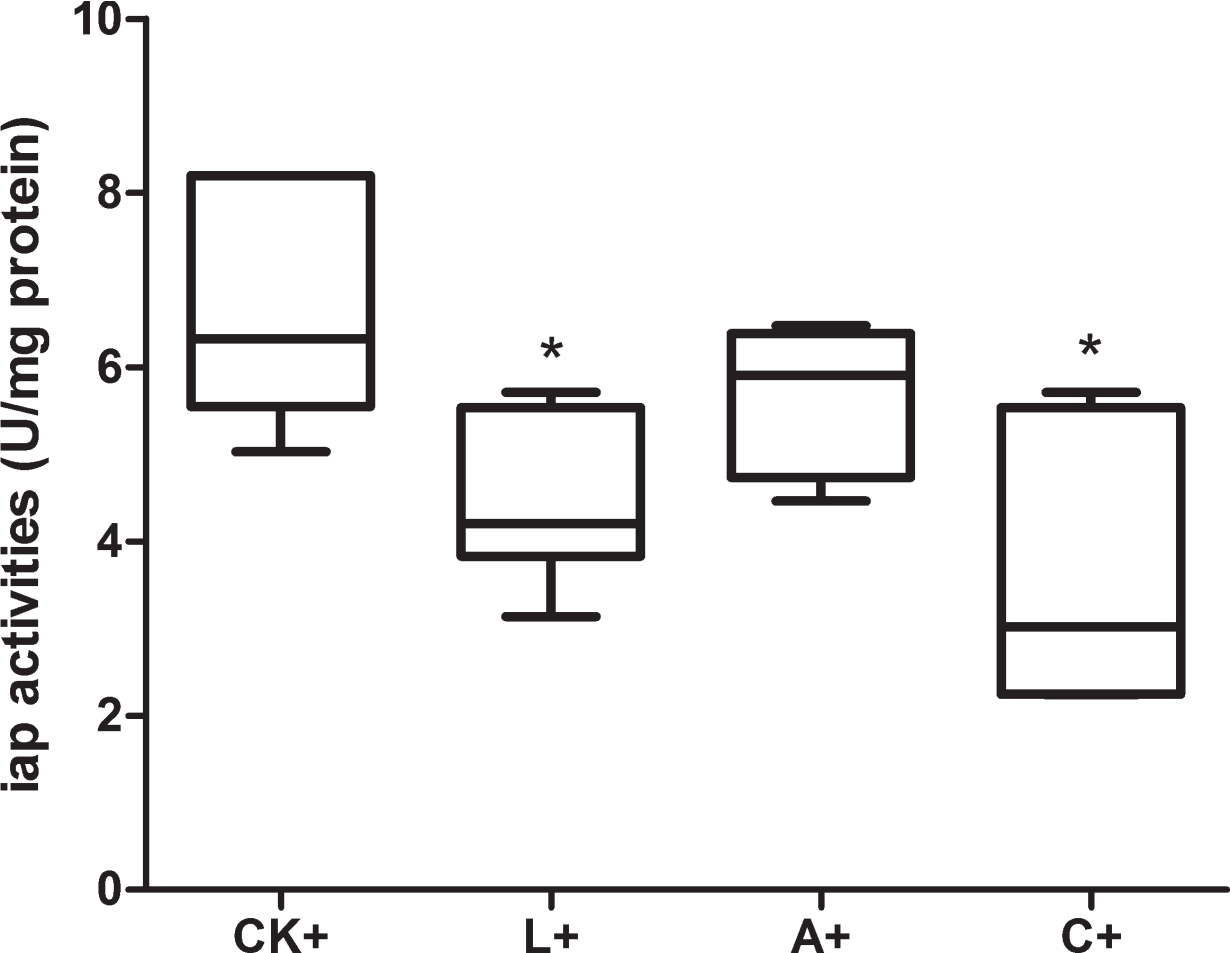
Intestinal (foregut) alkaline phosphatase activity (IAP) of tilapia fed different experimental diets under *Aeromonas hydrophila* infection at 24 h. CK+, fish dietary control diet and immersed by *A. hydrophila* NJ‐1; *Means significantly different (*P* < 0.05) compared to CKL, fish dietary *Lactobacillus plantarum* and immersed by *A. hydrophila* NJ‐1; A+, fish dietary AHL lactonase and immersed by *A. hydrophila* NJ‐1; C+, fish dietary *L. plantarum* and AHL lactonase and immersed by *A. hydrophila* NJ‐1.

IAP activities of tilapia 24 h post *A. hydrophila* challenge were significantly (*P *<* *0.05) lower in groups L+ and C+ compared to CK+, but no difference was observed in the A+ group(*P *>* *0.05) (Fig. 7). IAP is important in detoxifying the endotoxin component lipopolysaccharides (LPS) by dephosphorylation (Bates et al. 2007). The IAP activity was positively correlated with the number of *A. hydrophila* cells in the fish intestine challenge. Therefore, lower IAP activity after challenge indicates improved resistance of fish against pathogen (Liu et al. 2016). These results indicated that the number of *A. hydrophila* adhered to the gut was lower in L+ and C+ groups.

### Intestinal stress and cytokine genes expression

**Figure 8:**
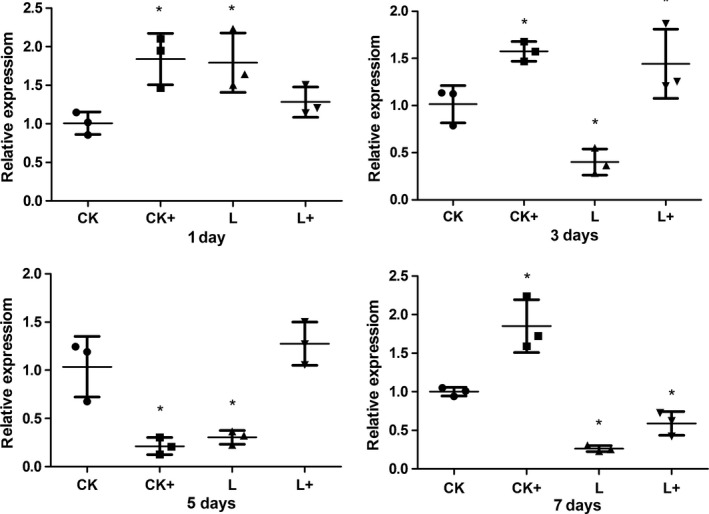
Intestinal (foregut) *hsp70* in tilapias fed *Lactobacillus plantarum* under *Aeromonas hydrophila* NJ‐1 infection or not at the end of 7 feeding days (*n* = 3). *Means significantly different (*P* < 0.05) compared to CK. The statistics was done per day. Ck and CK+, fish dietary control diet and immersed by *A. hydrophila* NJ‐1; L and L+, fish dietary *Lb. plantarum* and immersed by *A. hydrophila* NJ‐1.

Intestinal cytokine genes expression was evaluated only in the dietary *Lb. plantarum* treatments based on the results of Cao et al. (2014). *A. hydrophila* challenge generally up‐regulated (*P *<* *0.05) the expression of *hsp70* at day 1, 3, and 7 except that a down‐regulation (*P *<* *0.05) of *hsp70* expression was observed at day 5 in group CK+, suggesting the stress response upon *A. hydrophila* infection. Dietary *Lb. plantarum* (group L) significantly (*P *<* *0.05) reduced the expression of *hsp70* post the acute response phase (at day 3, 5, and 7; *P *<* *0.05). Compared to CK+, the expression of *hsp70* in group L+ was down‐regulated (*P *<* *0.05) at day 1 and 7, while an up‐regulation was observed at day 5 and no significant difference in *hsp70* expression was observed at day 3 (Fig. 8). These results indicated that *Lb. plantarum* could alleviate intestinal stress response.

**Figure 9:**
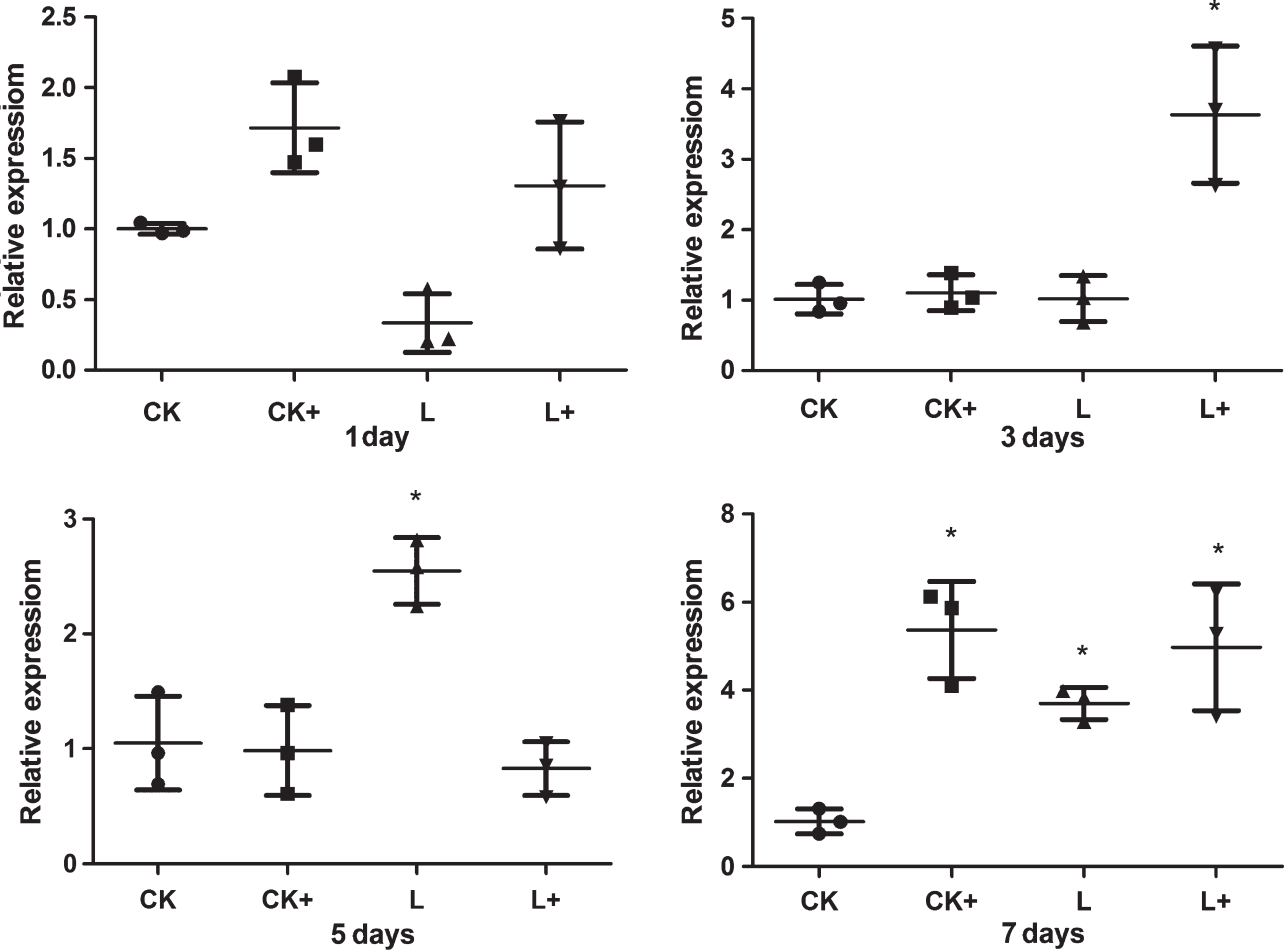
Intestinal (foregut) *il‐β* gene expression in tilapias fed *Lactobacillus plantarum* under *Aeromonas hydrophila* NJ‐1 infection or not at the end of 7 feeding days (*n* = 3). *Means significantly different (*P* < 0.05) compared to CK. The statistics was done per day. Ck and CK+, fish dietary control diet and immersed by *A. hydrophila* NJ‐1; L and L+, fish dietary *Lb. plantarum* and immersed by *A. hydrophila* NJ‐1.

**Figure 10:**
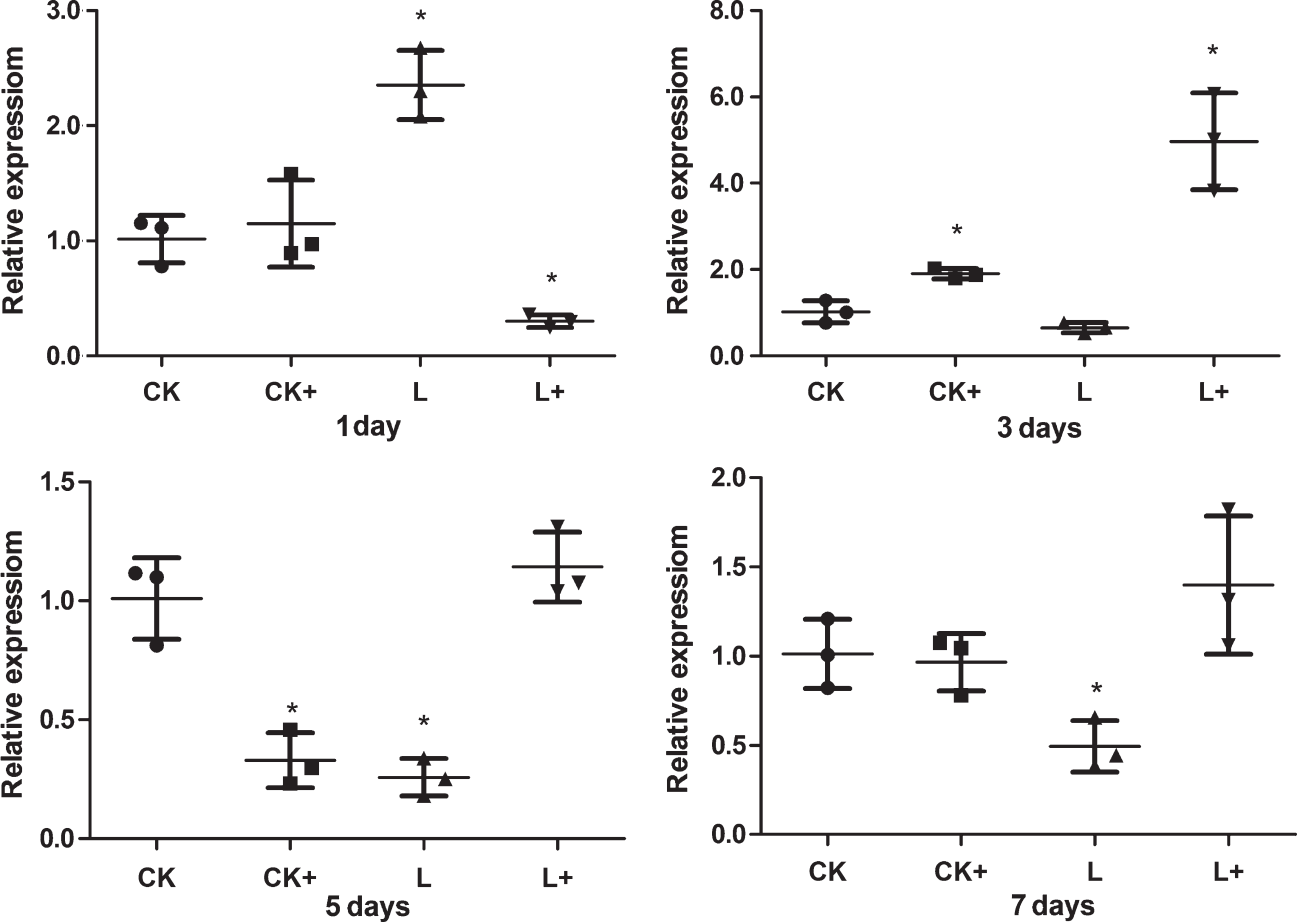
Intestinal (foregut) *tnf‐α* gene expression in tilapias fed *Lactobacillus plantarum* under *Aeromonas hydrophila* NJ‐1 infection or not at the end of 7 feeding days (*n* = 3). *Means significantly different (*P* < 0.05) compared to CK. The statistics was done per day. Ck and CK+, fish dietary control diet and immersed by *A. hydrophila* NJ‐1; L and L+, fish dietary *Lb. plantarum* and immersed by *A. hydrophila* NJ‐1.

**Figure 11:**
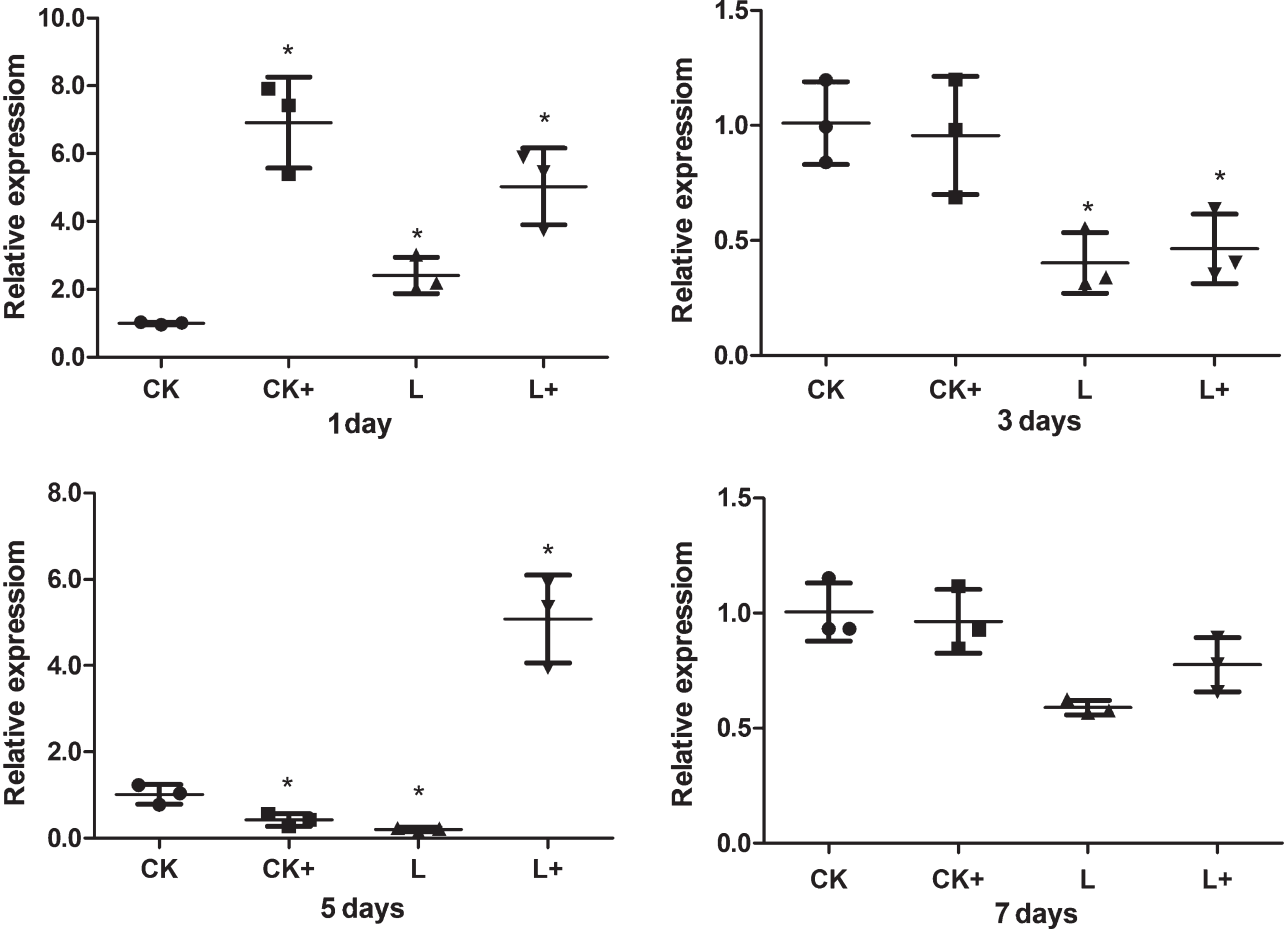
Intestinal (foregut) *tgf‐β* gene expression in tilapias fed *Lactobacillus plantarum* under *Aeromonas hydrophila* NJ‐1 infection or not at the end of 7 feeding days (*n* = 3). *Means significantly different (*P* < 0.05) compared to CK. The statistics was done per day. Ck and CK+, fish dietary control diet and immersed by *A. hydrophila* NJ‐1; L and L+, fish dietary *Lb. plantarum* and immersed by *A. hydrophila* NJ‐1.

*Aeromonas hydrophila* challenge up‐regulated (*P *<* *0.05) the expression of *il‐1β* gene at day 7 (group CK+). *Lactobacillus plantarum* also induced higher expression of *il‐1β* at day 5 and 7 (group L). In treatment group L+, the expression of *il‐1β* was significantly (*P *<* *0.05) increased at day 3 and 7 (Fig. 9). Meanwhile, dietary *Lb. plantarum* induced lower expressions of *tnf‐α* gene post the acute response phase (at day 5 and day 7; Fig. 10). In the case of *tgf‐β* gene expression, *A. hydrophila* challenge up‐regulated its expression at day 1, but down‐regulated the expression at day 5 (*P *<* *0.05). Dietary *Lb. plantarum* induced higher expression of *tgf‐β* gene at day 1, but lower expressions at day 3 and 5. In group L+, expression level of *tgf‐β* gene was up‐regulated at day 1 and 5 (Fig. 11).

## Discussion

*Lactobacillus* sp. is widely used in aquaculture for disease prevention (Verschuere et al. 2000; Ringø et al. 2010). As the importance of quorum sensing in virulence development of pathogenic bacteria is well known, disruption of quorum sensing was suggested as anti‐infective strategy (Finch et al. 1998). Quorum‐quenching enzymes have been investigated to disrupt the quorum sensing of pathogens and prevent the bacterial disease (Cao et al. 2012). Here, we first studied the combination of the two biological approaches; probiotics and quorum‐quenching enzymes have been as the control strategy in an ex vivo experiment.

In this study, the IAP activity 24 h after challenge was used to evaluate resistance of tilapia against *A. hydrophila* as IAP activity was positively correlated with the numbers of *A. hydrophila* cells in the fish intestine after challenge (Liu et al. 2016). In this study, IAP activities were significantly reduced in L+ and C+ groups, indicating enhanced resistance, probably related to adhesion competition of *Lb. plantarum* and *A. hydrophila* in the tilapia intestine, in accordance to that revealed by Ren et al. (2013). However, IAP activities of fish treated with AHL lactonase were not significantly influenced, suggesting that supplementation of AHL lactonase alone was not efficient to control *A. hydrophila* infection in tilapia though its prevent effects widely validated (Zhang et al. 2011; Cao et al. 2012; He et al. 2013).

In a previous study, Ren et al. (2013) revealed that *A. hydrophila* caused intestinal epithelial cell damage in the foregut of hybrid tilapia in an ex vivo exposure study. Clear differences in the intestinal histology were observed between fish exposed to *A. hydrophila* and *Lb. plantarum* strains (L *vs*. CK+). Damaged appearances were detected in CK+ group, but normal appearances were observed in the L and C group. Notably, the number of goblet cell increased in group L, A, and C. Intestinal goblet cell is a major immune cell (Johansson and Hansson 2014), and several studies have shown that diets and gut microbiota affects the numbers and secretory activity of goblet cell (McCracken et al. 1995; Sharma and Schumacher 1995a,b; Sharma et al. 1995). In this study, exogenous feed additives, AHL lactonase and *Lb. plantarum* increased the number of goblet cells when fish were not infected. Meanwhile, both *Lb. plantarum* and AHL lactonase modulate the microbial gut communities; similarity coefficient of 0.76 (marginally different) and 0.42 (significantly different) compared to the control, respectively (Ren et al. 2013; Cao et al. 2014), might responsible for the increased goblet cell numbers. Under infection conditions, all groups revealed increased goblet cells especially in the CK+ treatment. Increased goblet cells production has also been revealed in Arctic charr (*Salvelinus alpinus* L.) exposed to *Aeromonas salmonicida* in an in vivo study (Lødemel et al. 2001). Besides the goblet cells, multiple damage appearances were observed in intestine of group CK+. Dietary *Lb. plantarum* or/and AHL lactonase obviously reduced the extent of intestine damage, to a level similar as control fed fish, not exposed to *A*. *hydrophila*. Considering the negative effects in both single use and combination with *Lb. plantarum* caused shorter microvilli length and lower microvilli, the single use of dietary *Lb. plantarum* seems a better control strategy against *A. hydrophila* infection in tilapia.

In this study, heat‐shock protein *hsp70* was chosen as a stress marker because *hsp70* expression has been reported to be related to stressful factors in aquaculture, such as climate, water quality, diet, disease, and density (Rollo et al. 2006). Different treatments caused higher expression level of *hsp70* at day 1. Lower intestinal *hsp70* gene expression in fish fed diet supplemented with *Lb. plantarum* was observed at day 3, 5, and 7, indicating that dietary *Lb. plantarum* lowered stress response in vivo, in contrast to the results of Ren et al.(2013) revealing that *Lb. plantarum* induced the expression of *hsp70* in the anterior intestine of tilapia in an ex vivo experiment. Interestingly, the reduced expression of *hsp70* corresponded to the improved microvilli length under infection condition (Table 4). Cytokines are important factors in nonspecific immune system in fish (Magnadóttir 2006). The expression level of inflammatory cytokine genes of fish in different treatments were irregularly changed in this study. It might be due to the time course of immune and inflammatory responses. Nonspecific immune response is rapidly triggered by infectious pathogens or stresses, but would be suppressed in the later phase of a chronic challenge (Tort 2011). The expression level of *il‐1β* and *tnf‐α* were generally not influenced after infected with *A. hydrophila* (group C+) suggesting the suppressed immune response was not detected in 7 days. Further studies will include earlier sampling time points (0–12 h) and a long‐term infection to clarify these results. Expression level of *il‐1β* showed no different to the control group after fed with *Lb. plantarum* (group L) at day 1 and 3, but higher expressions of *il‐1β* were present in group L+. These indicated *Lb. plantarum* proved the nonspecific immune response during the a chronic challenge.

AHL lactonase has been reported to elevate the expression of *hsp70* and pro‐inflammatory cytokine genes (*il‐1β* and *tnf‐α*) (Cao et al. 2014). In addition, *il‐1β* and *tnf‐α* contributed to death of enterocytes (De Plaen 2013), which might contribute to the lower microvilli length and density in group A and lower microvilli length in group C. Considering the gut microvilli length in the C treatment, single use of *Lb. plantarum* strain JCM1149 seems to be a better control strategy against *A. hydrophila* infection in tilapia.

## Conflict of Interest

None declared.
